# Reactive Plasma N-Doping of Amorphous Carbon Electrodes: Decoupling Disorder and Chemical Effects on Capacitive and Electrocatalytic Performance

**DOI:** 10.3389/fchem.2020.593932

**Published:** 2020-11-03

**Authors:** Md. Khairul Hoque, James A. Behan, James Creel, James G. Lunney, Tatiana S. Perova, Paula E. Colavita

**Affiliations:** ^1^Centre for Research on Adaptive Nanostructures and Nanodevices (CRANN) and SFI Research Centre for Advanced Materials and BioEngineering Research (AMBER) Research Centres, School of Chemistry, Trinity College Dublin, Dublin, Ireland; ^2^School of Physics, Trinity College Dublin, Dublin, Ireland; ^3^School of Engineering, Trinity College Dublin, College Green, Dublin, Ireland

**Keywords:** carbon, electrocatalysis and functionalized/modified electrodes, metal-free, amorphous carbon (a-C), nitrogen plasma activation, nitrogen plasma (nitridation)

## Abstract

Nitrogen-free amorphous carbon thin films prepared via sputtering followed by graphitization, were used as precursor materials for the creation of N-doped carbon electrodes with varying degrees of amorphization. Incorporation of N-sites was achieved via nitrogen plasma treatments which resulted in both surface functionalization and amorphization of the carbon electrode materials. X-ray photoelectron spectroscopy (XPS) and Raman spectroscopy were used to monitor composition and carbon organization: results indicate incorporation of predominantly pyrrolic-N sites after relatively short treatment cycles (5 min or less), accompanied by an initial etching of amorphous regions followed by a slower process of amorphization of graphitized clusters. By leveraging the difference in the rate of these two processes it was possible to investigate the effects of chemical N-sites and C-defect sites on their electrochemical response. The materials were tested as metal-free electrocatalysts in the oxygen reduction reaction (ORR) in alkaline conditions. We find that the introduction of predominantly pyrrolic-N sites via plasma modification results in improvements in selectivity in the ORR, relative to the nitrogen-free precursor material. Introduction of defects through prolonged plasma exposure has a more pronounced and beneficial effect on ORR descriptors than introduction of N-sites alone, leading to both increased onset potentials, and reduced hydroperoxide yields relative to the nitrogen-free carbon material. Our results suggest that increased structural disorder/heterogeneity results in the introduction of carbon sites that might either serve as main activity sites, or that enhance the effects of N-functionalities in the ORR via synergistic effects.

## Introduction

Carbon electrode materials are ubiquitous in energy applications due to several important desirable characteristics, namely, their earth-abundance, low cost, good scalability and processability, high conductivity, versatile morphology, tunable microstructure and crystallinity, and good mechanical properties (Georgakilas et al., 2015; Salanne et al., 2016; Dou et al., 2019). Carbon materials have been widely explored for the fabrication of electrochemical capacitors, batteries and fuel cells (Inagaki et al., 2010; Marom et al., 2011; Dou et al., 2019) and are expected to continue to play an important role in future energy technologies as the world transitions to a more sustainable economy with reduced reliance on fossil fuels (Salanne et al., 2016; Perathoner and Centi, 2018; Dou et al., 2019; Domìnguez et al., 2020).

The incorporation of N-sites has emerged as an effective strategy for enhancing and expanding on the electrochemical performance of carbon materials. Nitrogenated carbons and nanocarbons have become important in the area of non-precious and metal-free electrocatalysts for fuel cell applications (Wong et al., 2013; Gong et al., 2015), while also being proposed as materials for supercapacitors in energy storage (Deng et al., 2016; Salanne et al., 2016; Yang and Zhou, 2017). As a consequence, nitrogen incorporation strategies have been explored for a range of carbon electrode materials, including carbon nanotubes (Gong et al., 2009; Tang et al., 2009), graphene (Wei et al., 2009; Qu et al., 2010), graphite (Zhou et al., 2010), nanofibers (Maldonado and Stevenson, 2005) and amorphous/non-crystalline carbons (Wang et al., 2010; Zhang et al., 2015; Yamamoto et al., 2018; Dou et al., 2019). Nitrogen incorporation results in complex and interrelated effects on the electrochemical properties of carbons, resulting from a combination of electronic (Behan et al., 2017), chemical and structural changes (Serp and Figueiredo, 2009; Dou et al., 2019). Functional N-sites such as pyridinic-N (N_Py_), pyrrolic-N (N_Pr_), graphitic-N (N_G_), and N-oxides (N_Ox_) can be incorporated into the carbon structure (Serp and Figueiredo, 2009) resulting in reactive centers that can significantly affect interfacial interactions with redox species and intermediates (Choi et al., 2014; Guo et al., 2016; Wu et al., 2017; Behan et al., 2018, 2019b) and, consequently, electrocatalytic activity. Nitrogen sites can also affect the electronic properties of carbon electrodes, as they might serve as both n-type or p-type dopants (Zhao et al., 2011; Ma et al., 2018), and can increase carbon conductivity and metallic character (Robertson, 2002; Choi et al., 2014; Behan et al., 2017; Hoque et al., 2019). Furthermore, the role of nitrogen atoms as impurities and the experimental methods used for its incorporation can affect the organization of the carbon scaffold, introducing voids, vacancies, defects and non-graphitizable regions that have dramatic effects on both capacitive and faradaic responses (Zhou et al., 2010; Legrain et al., 2015; Salanne et al., 2016; Stamatin et al., 2016; Zhang et al., 2016; Behan et al., 2017, 2019a; Ng et al., 2018; Dou et al., 2019; Hoque et al., 2019; Jia et al., 2019; Saurel et al., 2019). The importance of improving our understanding and control over the interplay among disorder, functional sites, and electrochemical performance has been highlighted in the recent literature as one of the frontiers in the development of new carbon-based materials for energy technologies (Legrain et al., 2015; Zhang et al., 2016; Bommier et al., 2019; Dou et al., 2019; Saurel et al., 2019). Therefore, there is great interest in developing approaches for achieving control over nanostructuring and chemical functionality to enable a better understanding of structure-function correlations, and novel strategies for the synthesis of smart carbons with tailored ad-/chemisorption sites at heteroatom and carbon defect centers (Salanne et al., 2016; Dou et al., 2019).

In this work we have used nitrogen-free amorphous carbon thin films as precursor materials for the creation of carbon electrodes with constant N/C composition that possess varying degrees of amorphization. Incorporation of N-sites was achieved via low-energy reactive plasma; plasma/${\text{N}}_{2^{+}}$ bombardment methods have previously been used to introduce isolated defects in highly ordered pyrolytic graphite (Kondo et al., 2012), however their effect on amorphous or partially graphitized systems remains unexplored. Our results show that plasma methods enable a fast, self-limiting chemical modification of the disordered carbon surface in parallel with an increase in amorphization degree that occurs at a lower rate. By leveraging the difference in the rate of these two processes it was possible to decouple the effects of chemical N-sites and C-defect sites on the electrochemical response of these materials and understand the role of the two contributions on their capacitive and faradaic response. X-ray photoelectron spectroscopy (XPS) and Raman spectroscopy were used to monitor composition and carbon organization. Electrochemical impedance spectroscopy (EIS) was used to characterize their capacitive response. Finally, the materials were tested as metal-free electrocatalysts in the oxygen reduction reaction (ORR) under alkaline conditions to identify correlations between composition and ORR activity descriptors. We find that the introduction of predominantly pyrrolic-N sites via plasma modification results in improvements in activity and selectivity in the ORR, relative to the nitrogen-free precursor material. Introduction of defects through prolonged plasma exposure has a more pronounced and beneficial effect on ORR activity descriptors than introduction of N-sites alone, that cannot be justified on the basis of changes in N-site composition or microroughness. We propose that increased structural disorder/heterogeneity results in the introduction of carbon sites that could serve as the main sites or enhance the activating effects of N-functionalities in the ORR.

## Materials and Methods

### Chemicals and Materials

Tetrabutylammonium hexafluorophosphate (TBAPF_6_) (≥99.0%, electrochemical analysis), acetonitrile (MeCN, 99.8%, anhydrous), sulfuric acid (95–97%), hydrogen peroxide (>30% w/v), potassium hydroxide (semiconductor grade pellets, 99.99%) were purchased from Sigma Aldrich and used without further purification. Glassy carbon (GC) disks (HTW Sigradur® radius 2.5 mm) and B-doped Si wafers (MicroChemicals; resistivity 5–10 Ω cm) were used as substrate materials for carbon deposition.

### Substrate Preparation

GC disks were used as deposition substrates for all electrochemical experiments. Disks were polished as previously described (Hoque et al., 2019): first, using 1,200 grit sandpaper and 1 μm alumina slurry (Buehler); second, using 1 μm slurry on nylon paper; finally, using 0.3 and 0.05 μm slurries on MicroCloths® pads (Buehler) to a mirror finish. Between polishing steps, GC disks were sonicated for 20 min in Millipore water to avoid cross-contamination between alumina particle sizes. Clean disks were mounted in a custom-made Teflon® holder prior to sputter deposition of amorphous carbon electrodes (*vide infra*). Si wafers were used for all spectroscopic characterization experiments. Wafers were cleaned in piranha solution (3:1 H_2_SO_4_:H_2_O_2_; *CAUTION piranha solutions are explosive in contact with organics*), rinsed with plenty of Millipore water and finally dried with Ar prior to deposition.

### Deposition of Carbon Electrode Materials

**Figure 1:**
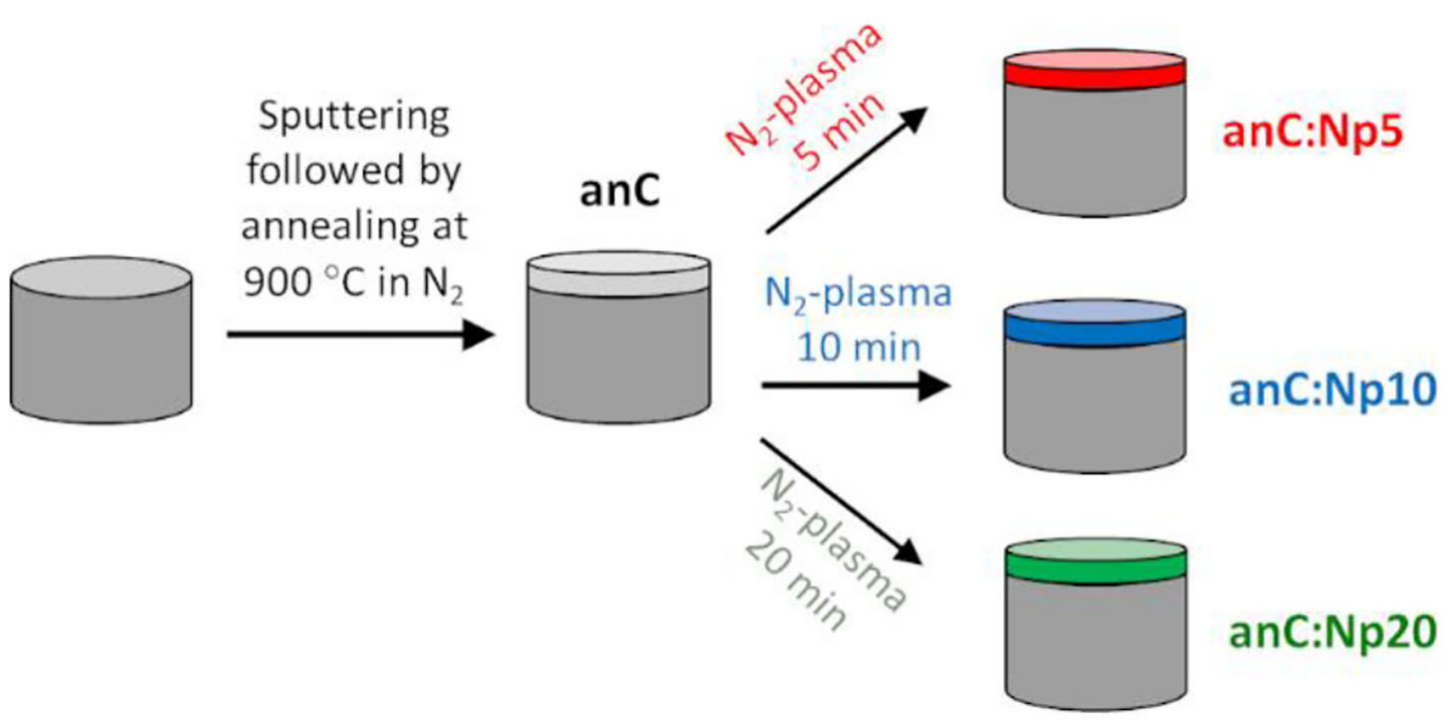
Schematic representation of carbon deposition and modifications used in our experiments.

Nitrogen-free amorphous carbon thin film materials were synthesized via magnetron sputtering followed by post-deposition thermal graphitization (Figure 1). DC-magnetron sputtering from a graphite target (99.999%) was carried out in a deposition chamber (Torr International) at base pressure <2 × 10^−6^ mbar; carbon was sputtered for 40 min onto GC disks at an Ar pressure of 2–7 × 10^−3^ mbar achieved at a flow of 50 mL min^−1^, as previously reported (Cullen et al., 2012; Behan et al., 2017). The resulting carbon films are topographically smooth, with thickness of *ca*. 70 nm and with high graphitic content (>80% sp^2^-centers) (Cullen et al., 2012; Behan et al., 2017). Sputtered electrodes were then graphitized in a tube furnace under N_2_ flow for 1 h at 900°C, resulting in graphitized amorphous carbon film electrodes with smooth topography (Behan et al., 2018). These materials were used as precursors for the creation of nitrogenated carbons via plasma surface modifications. Surface nitrogenation was carried out in a custom-built 25 mm O.D. quartz plasma chamber with a base pressure of 10–25 mbar, equipped with an induction coil connected to an RF generator (13.56 MHz, ENI). N_2_ gas was fed at 20 mL min^−1^ using a mass flow controller (Brooks Instruments), maintaining a pressure of 160 mbar during plasma treatments. The graphitized nitrogen-free electrodes were exposed to the N_2_-plasma at ~10 W RF coupled power for 5, 10, and 20 min.

### Characterization

XPS was performed in a VG Scientific ESCAlab Mk II system (<2 × 10^−8^ mbar), using Al Kα X-rays (1486.6 eV); core-level spectra were collected with analyzer pass energy of 20 eV and survey spectra were collected with analyzer pass energy of 200 eV. Peaks were fitted with Voigt functions after Shirley background subtraction using commercial software (CasaXPS); at.% compositions were obtained from peak area ratios after correction by Scofield relative sensitivity factors (C = 1.0, N = 1.8, O = 2.93). Raman spectra were measured in backscattering configuration using a Renishaw 1,000 micro-Raman system equipped with an Ar^+^ laser with 488 nm excitation. The incident beam was focused by a Leica microscope with a 50× magnification objective and short-focus working distance; incident power was kept <2 mW to avoid sample damage. Spectra were baseline corrected using commercial software prior to analysis (Wire 3.2).

Electrochemical studies were carried using a three-electrode cell thermostated at 25°C and controlled by a Metrohm Autolab AUT50324 potentiostat. A Hydroflex hydrogen electrode (Gaskatel) and an Ag/Ag^+^ electrode (IJCambria) were used as references in aqueous and organic electrolyte, respectively. The Ag/Ag^+^ reference electrode was prepared using 1.0 mM AgNO_3_ in MeCN, yielding a reference potential of 0.320 V vs. SHE, determined as described in previous work (Hoque et al., 2019). The carbon disk electrodes were mounted in a Teflon disk holder (Pine Instruments) and used as working electrodes; all contacts were confirmed to be ohmic with <8 Ω resistance. Graphite rods (Goodfellow) were used as counter electrodes. Cyclic voltammetry (CV) was carried out in 0.1 M TBAPF_6_ in MeCN, at 50 mV s^−1^ using iR compensation. Electrochemical impedance spectroscopy (EIS) was recorded in the range 0.1–10^4^ kHz using a 10 mV AC amplitude at open circuit potential (OCP). The equivalent series capacitance was obtained from Mott-Schottky plots (NOVA software) collected at the frequency indicated in each plot, with steps of 0.05 V; 300 s equilibration time was allowed between potential steps. The specific capacitance was obtained via normalization by the geometric area of the electrodes; this was determined in 0.1 M TBAPF_6_/MeCN using a reference polished GC disk to account for capillary wetting within the Teflon shielding in MeCN (De Levie, 1965) and a specific capacitance value of 30 μF cm^−2^ reported for GC in TBA^+^/MeCN (Kim et al., 2010). Studies of the oxygen reduction reaction were carried out using a rotating disk electrode (RDE) and a rotating ring-disk electrode (RRDE) tip (Pine Instruments). The electrochemical cell was cleaned using piranha solution; working electrodes were first cycled between 0.05 and 1.1 V_RHE_ (potential vs. RHE, i.e., reversible hydrogen electrode) in Ar-saturated 0.1 M KOH (20 cycles). Then, ORR polarization curves at 50 mV s^−1^ were obtained in O_2_-saturated 0.1 M KOH electrolyte under rotation. CVs in Ar-saturated 0.1 M KOH were used to subtract the capacitive current contribution, while current densities were calculated by normalizing the current by the geometric area. All voltammograms were obtained with iR compensation using commercial software (NOVA); the uncompensated resistance was determined via EIS prior to each experiment. RRDE experiments were carried out by holding the Pt ring insert at a constant potential of 1.2 V_RHE_; the peroxide yield was calculated as $H_{2}O_{2}\%=100\times 2\left(\frac{I_{R}/N}{I_{D}+I_{R}/N}\right)$, where *I*_*R*_ and *I*_*D*_ denote the ring and disk currents, respectively, and *N* is the collection efficiency determined experimentally (*N* = 0.26) (Behan et al., 2019b). The number of electrons was calculated according to *n* = 4*I*_*D*_/(*I*_*D*_ + *I*_*R*_/*N*).

## Results and Discussion

### Chemical and Structural Characterization of anC:N Electrode Materials

Nitrogen-free electrodes were prepared in the form of thin solid films via magnetron sputtering from a graphite target onto GC disk electrodes, as shown schematically in Figure 1. Films were subsequently annealed under N_2_ atmosphere for 1 h at 900°C resulting in graphitized amorphous carbon electrodes (anC) (Behan et al., 2018), that were used as the precursor material for nitrogen functionalization. The anC electrodes were modified in a reactive N_2_-plasma for 5, 10, and 20 min resulting in electrode materials denoted anC:Np5, anC:Np10, and anC:Np20, respectively.

**Figure 2:**
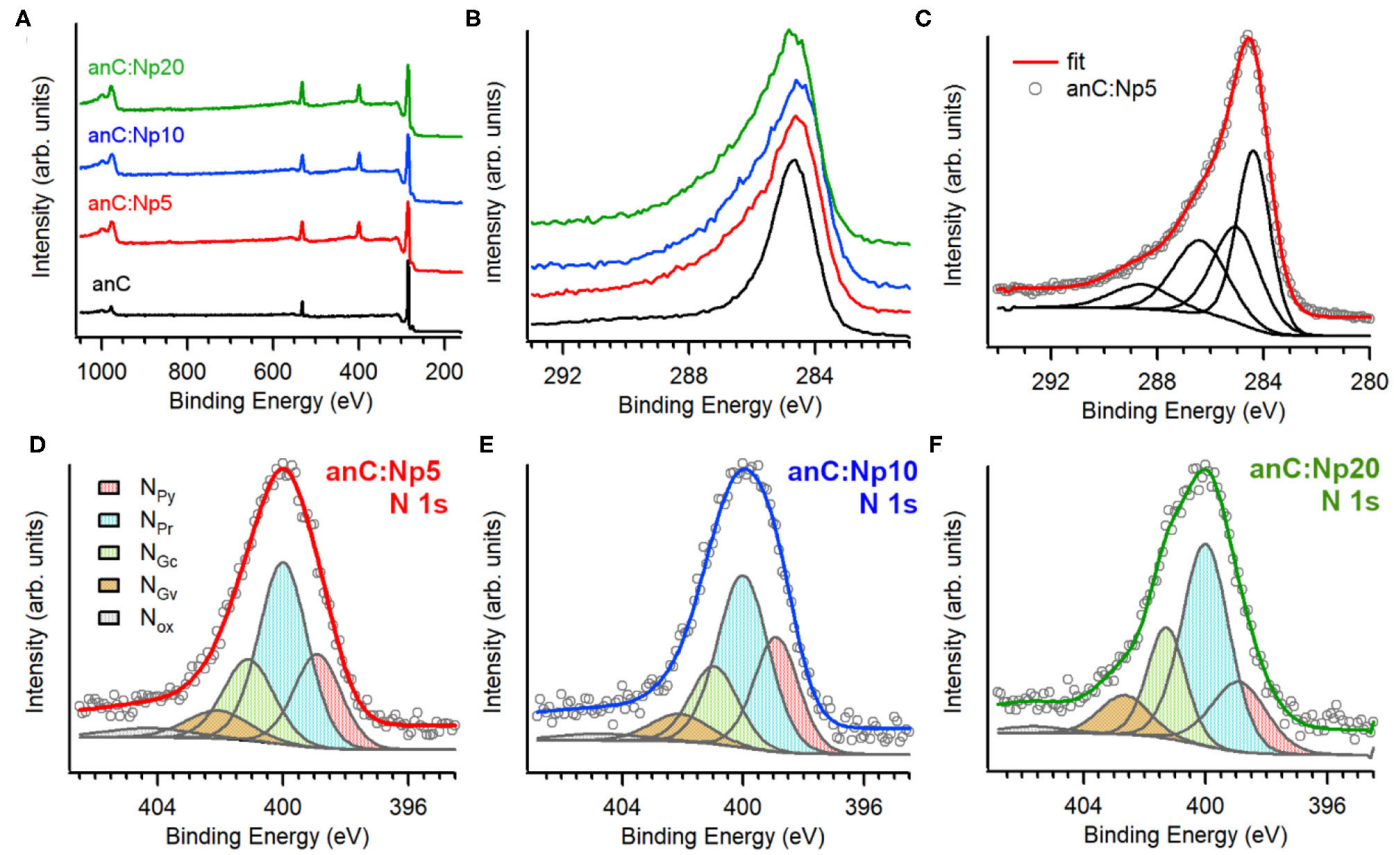
**(A)** Survey scans and **(B)** high resolution C 1s spectra of anC, anC:Np5, anC:Np10, and anC:Np20 electrodes; **(C)** shows details of the C 1s decomposition for anC:Np5 into four main contributions. High resolution N 1 s spectra of **(D)** anC:Np5, **(E)** anC:Np10, and **(F)** anC:Np20 together with their best-fit decompositions into N_Py_ (398.9 eV), N_Pr_ (400 eV), N_Gc_ (401 eV), N_Gv_ (402.5 eV), and N_Ox_ and satellites (>403 eV). Individual peak contributions are offset from the spectral envelope for the sake of clarity.

Figure 2A shows XPS survey spectra of anC, anC:Np5, anC:Np10, and anC:Np20 electrodes. Spectra show the characteristic peaks of C 1s, O 1s, and N 1s photoemissions at ~285, 532, and 400 eV, respectively. The N 1s peak is absent in the case of anC, the N-free precursor material, as discussed in detail in previous work from our group (Behan et al., 2018), however, it is clearly detectable after only 5 min exposure to the N_2_-plasma thus indicating that this treatment leads to functionalization of the carbon electrode with N-sites. The amount of nitrogen and oxygen incorporated into the films was calculated from the area ratios A_N1s_/A_C1s_ and A_O1s_/A_C1s_, respectively, after correction for the relative sensitivity factors; the at % composition thus calculated is reported in Table 1. The presence of oxygen likely originates from air exposure prior to characterization and residual water vapor in the quartz chamber. The compositional analysis shows that nitrogen and oxygen contents increase after 5 min of plasma treatment and plateau for longer plasma exposures. This suggests that the most significant changes in heteroatom content occur in <5 min under our reactive plasma conditions.

**Table 1:** C 1s fwhm values and at % composition of nitrogen-free and plasma treated carbon electrodes obtained from XPS analysis.

**Sample**	**O/C**	**N/C**	**C1s fwhm (eV)**
anC*^*a*^*	5.3%	–	1.6
anC:Np5	11%	16%	2.2
anC:Np10	11%	17%	2.4
anC:Np20	14%	17%	2.4

*a—Data from Behan et al. (2018)*.

Figure 2B shows a comparison of the high resolution C 1s peak of the carbon electrodes. All C 1s spectra show an asymmetric line characteristic of amorphous carbon materials with a maximum at *ca*. 284.5 eV, corresponding to the binding energy of sp^2^-centers in a graphitized scaffold (Díaz et al., 1996). Plasma treatments result in an increase in the full width at half maximum (fwhm) of the C 1s peak (Table 1), that is consistent with the introduction of heteroatoms and/or with changes in the degree of graphitization of the carbon structure (Maldonado et al., 2006; Behan et al., 2018). The C 1s peak was fitted using four main components (Behan et al., 2018) as shown in Figure 2C for anC:Np5: 284.4 eV corresponding to sp^2^-centers, 285.2 eV assigned to sp^3^-centers, 286.5 eV assigned to C-O/C-N groups, and 288.5 eV that can be attributed to C - O contributions. The spectral overlap between C–O and C–N peaks precludes a more detailed and unambiguous resolution of their contributions to the C 1s line (Perini et al., 2015; Behan et al., 2018). Best-fit results of C 1s spectra of all carbon electrodes are reported in Supporting Information.

Figures 2D–F show the high resolution N 1s spectra and best-fits for anC:Np5, anC:Np10, and anC:Np20 electrodes, respectively. All spectra are broad thus indicating that a mixture of N-functional groups results from plasma exposure. Spectra were fitted using five main contributions (Biniak et al., 1997; Rodil et al., 1999; Maldonado et al., 2006; Sharifi et al., 2012) corresponding to pyridinic-N (N_Py_, 398.9 eV), pyrrolic-N (N_Pr_, 400.0 eV), graphitic-center (N_Gc_, 400.9–401.3 eV), graphitic-valley (N_Gv_, 402.1–402.6 eV), and N-oxides (N_Ox_) and possibly π-π^*^ shake-up satellites at high binding energies (>403 eV). The relative contributions of these five components do not vary significantly with plasma exposure time (see Supporting Information): pyrrolic-N accounts for the largest contribution (*ca*. 45%) in all three nitrogenated materials, followed by graphitic-N (*ca*. 30%) and pyridinic-N (*ca*. 22%) sites. Overall, the XPS data indicates that N-functional group incorporation occurs rapidly, within 5 min of plasma exposure time, and that longer exposures do not affect either the N/C content or the distribution of N-functionalities.

**Figure 3:**
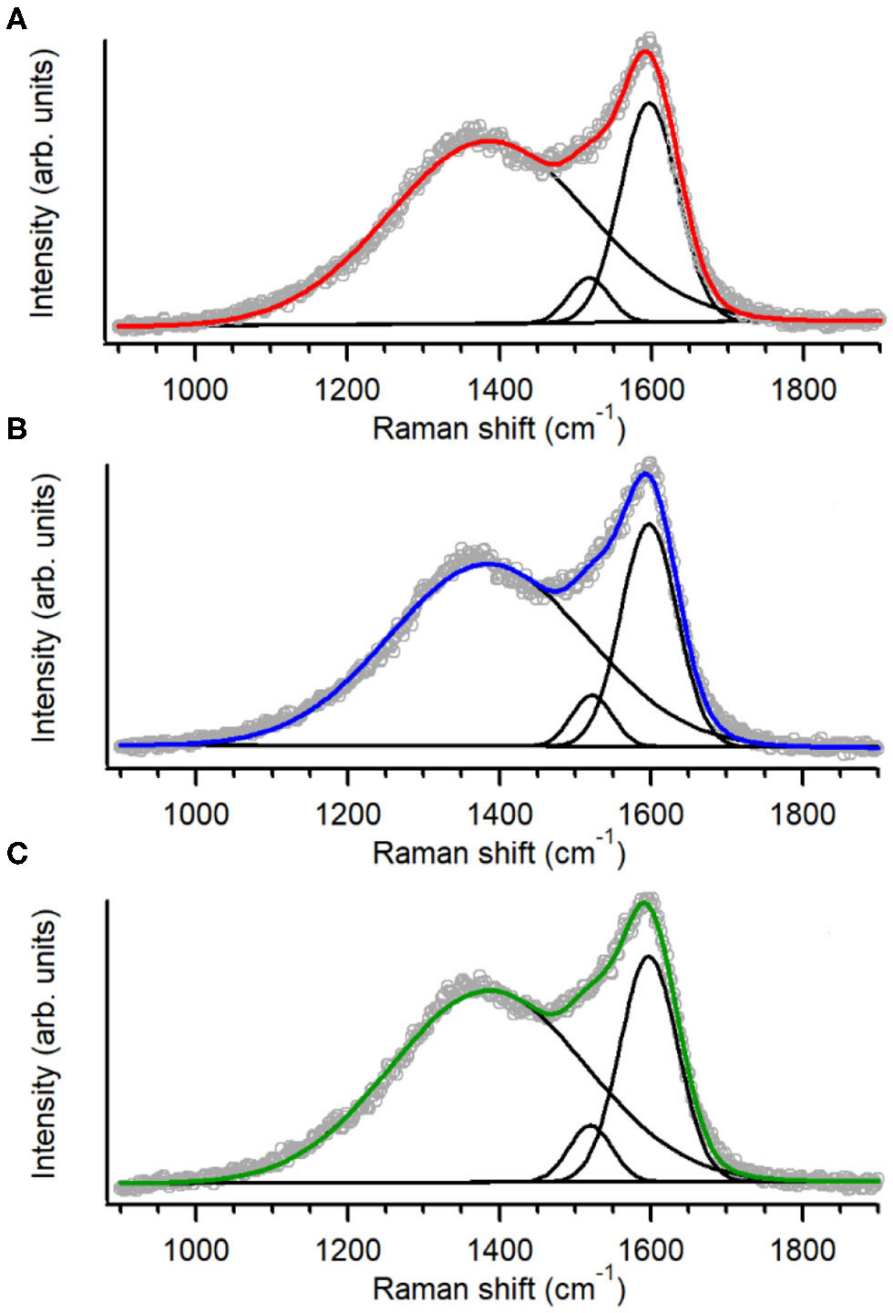
Raman spectra and peak best-fits of **(A)** anC:Np5, **(B)** anC:Np10, and **(C)** anC:Np20; spectra are normalized relatively to the G band intensity.

The structure of the carbon scaffold of the nitrogenated anC:N carbon materials was investigated via Raman spectroscopy; the Raman characterization of the nitrogen-free anC material has been reported elsewhere (Behan et al., 2018). Figures 3A–C show baseline-corrected Raman spectra in the 900–1,900 cm^−1^ range of anC:Np5, anC:Np10 and anC:Np20, respectively; spectra are shown after normalization by the main peak intensity to facilitate comparison. All spectra display the characteristic peaks assigned to the G and D bands at ~1,580 and 1,380 cm^−1^, respectively. The G peak is associated to stretching modes of carbon atoms in sp^2^-centers, in either rings or unsaturated chains. The D peak is associated to a breathing mode of sp^2^-centers in six-membered rings and it becomes Raman active if the symmetry of graphene clusters is broken due to the presence of defects (Ferrari and Robertson, 2000; Waidmann et al., 2001; Ferrari et al., 2003). Spectra were fitted using three Gaussian functions following previous methods (Laidani et al., 1997; Das et al., 2002; Behan et al., 2018): two functions were used to model G and D contributions, whereas the third one was introduced to model the A peak at *ca*. 1,510 cm^−1^. This third peak is ascribed to C-C stretching modes in the amorphous network that connects graphitic crystallites and that displays a mixture of three- and four-fold bonded carbon atoms (Laidani et al., 1997; Das et al., 2002).

Spectral parameters obtained from the fits are shown in Table 2; parameters for the nitrogen-free material are also reported for comparison. The results show that after plasma exposure there is an increase of the I_D_/I_G_ ratio and of the fwhm value of G and D peaks relative to the nitrogen-free anC materials that is indicative of increased defects in graphitic regions (Ferrari et al., 2003; Ferrari and Robertson, 2004; Sadezky et al., 2005; Martins Ferreira et al., 2010). The I_A_/I_G_ ratio decreases at first, relative to anC, likely due to rapid etching of amorphous regions connecting graphitized clusters. After longer times the I_A_/I_G_ ratio instead increases, indicating that prolonged plasma exposure results in amorphization of the carbon scaffold. The parameter trends are consistent with a process involving, first, an increase in graphene edges/defects through reactive etching and modification of amorphous regions interconnecting graphitized clusters, followed by further amorphization at the expense of ordered graphitic clusters.

**Table 2:** Raman spectral parameters for anC and anC:Np5, anC:Np10, and anC:Np20 electrode materials.

**Sample**	**I_**D**_/I_**G**_**	**I_**A**_/I_**G**_**	**G peak (cm**^****−1****^**)**		**D peak (cm**^****−1****^**)**	
			**position**	**fwhm**	**position**	**fwhm**
anC*^*a*^*	0.74	0.23	1,598	85	1,379	274
anC:Np5	0.83	0.20	1,596	89	1,386	308
anC:Np10	0.82	0.23	1,597	87	1,385	306
anC:Np20	0.85	0.25	1,597	89	1,386	303

*a—Data from Behan et al. (2018)*.

### Electrochemical Studies of anC:N Electrode Materials

The chemical and structural characterization of anC:N materials indicates that they possess similar functional groups, with predominantly pyrrolic-N groups at the surface resulting from plasma treatments. Raman spectroscopy indicates that plasma exposure leads to increased amorphization and defect density in the carbon material, thus offering a route to exploring the effect of disorder on the capacitive, and faradaic electrochemical response of nitrogenated materials with similar surface functionalities.

**Figure 4:**
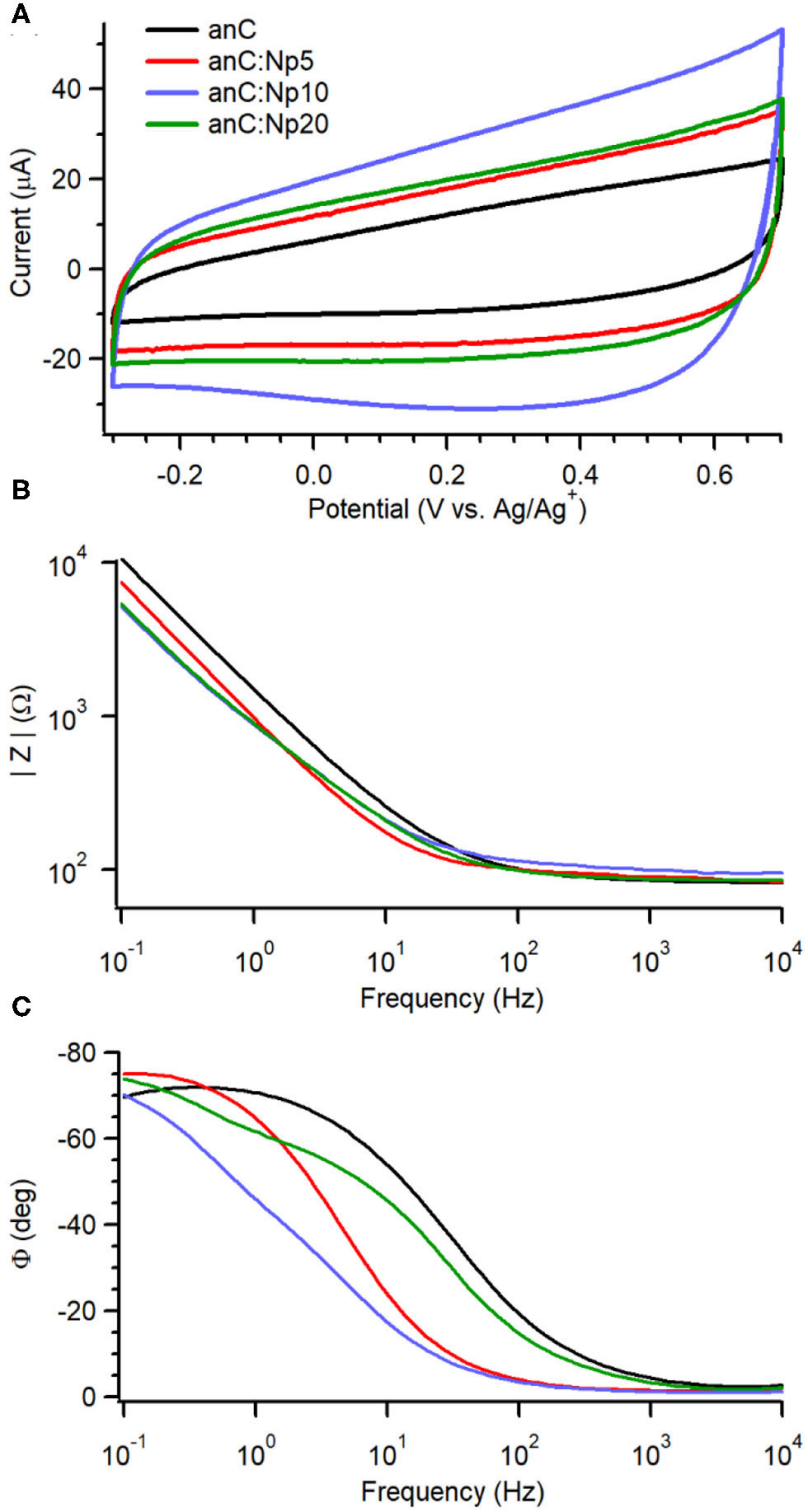
**(A)** Cyclic voltammograms of anC:Np5, anC:Np10, and anC:Np20 electrodes in Ar-saturated 0.1 M TBAPF_6_ in acetonitrile at 50 mV s^−1^; the CV of the nitrogen-free anC material is also shown for comparison. Impedance modulus **(B)** and phase **(C)** obtained from EIS data (Bode plots) in the same solution at open circuit potential (OCP −0.28–0.1 V vs. Ag/Ag^+^) are also shown for the electrode materials.

To investigate the effects of nitrogenation and amorphization on the capacitive properties we used an organic electrolyte of large ionic radii to minimize pseudo-capacitive contributions arising from redox functional groups at the carbon surface (Gerischer et al., 1987; Hahn et al., 2004; Wiggins-Camacho and Stevenson, 2009; Barranco et al., 2010; Vaquero et al., 2012; Hoque et al., 2019). Figure 4A shows cyclic voltammograms (CVs) in 0.1 M TBAPF_6_ solutions in acetonitrile at 50 mV s^−1^ for anC:Np5, anC:Np10 and anC:Np20 materials, together with that obtained for the nitrogen-free anC electrode under identical conditions. The CVs display the characteristic shape of a capacitive response, while no faradaic peaks are discernible within the chosen potential window. Larger capacitive currents were observed at nitrogenated electrodes than at anC.

EIS over the range 0.1–10^4^ Hz in 0.1 M TBAPF_6_ in acetonitrile reveals further details on the effect of plasma exposure on interfacial capacitance. Figures 4B,C display Bode plots of absolute impedance (|Z|) and phase angle (ϕ) at OCP (−0.28 to 0.05 V vs. Ag/Ag^+^) for plasma-treated anC:Np5, anC:Np10 and anC:Np20 electrodes; the Bode plot of the nitrogen-free anC precursor is also shown for comparison. The nitrogen-free anC material displays a response close to that of an equivalent RC circuit, with a capacitive contribution dominant at low frequency and a resistive response at high frequency; the values of ϕ ≈ −70° at 0.1 Hz indicates deviations from ideal capacitive behavior, which instead should yield a value of−90°. The short 5 min exposure to plasma results in a significant reduction in the |Z| value at low frequency, relative to the nitrogen-free anC precursor. Interestingly, the ϕ value also decreases to *ca*. −75° at 0.1 Hz, suggesting a response that is closer to that of a pure ideal capacitor (−90°). This is in agreement with Raman results which suggest that over short exposure times, nitrogenation might take place at the expense of amorphous surface regions. Further plasma exposure for 10 and 20 min leads to an additional decrease in |Z| value that plateaus between anC:Np10 and anC:Np20 electrodes. The phase plot shows evidence of an increase in the characteristic time constant in the order anC < anC:Np5 < anC:Np10 that is consistent with an increase in the interfacial capacitance, while for anC:Np20 it is possible to observe more pronounced deviations from ideality and evidence of at least a second partially resolved time constant. The EIS data therefore indicate that plasma treatments result in structural/electronic reorganizations of the carbon scaffold that progress over 5–20 min exposure times, despite the chemical N-functionalities remaining approximately constant at the carbon surface after only 5 min of exposure.

**Figure 5:**
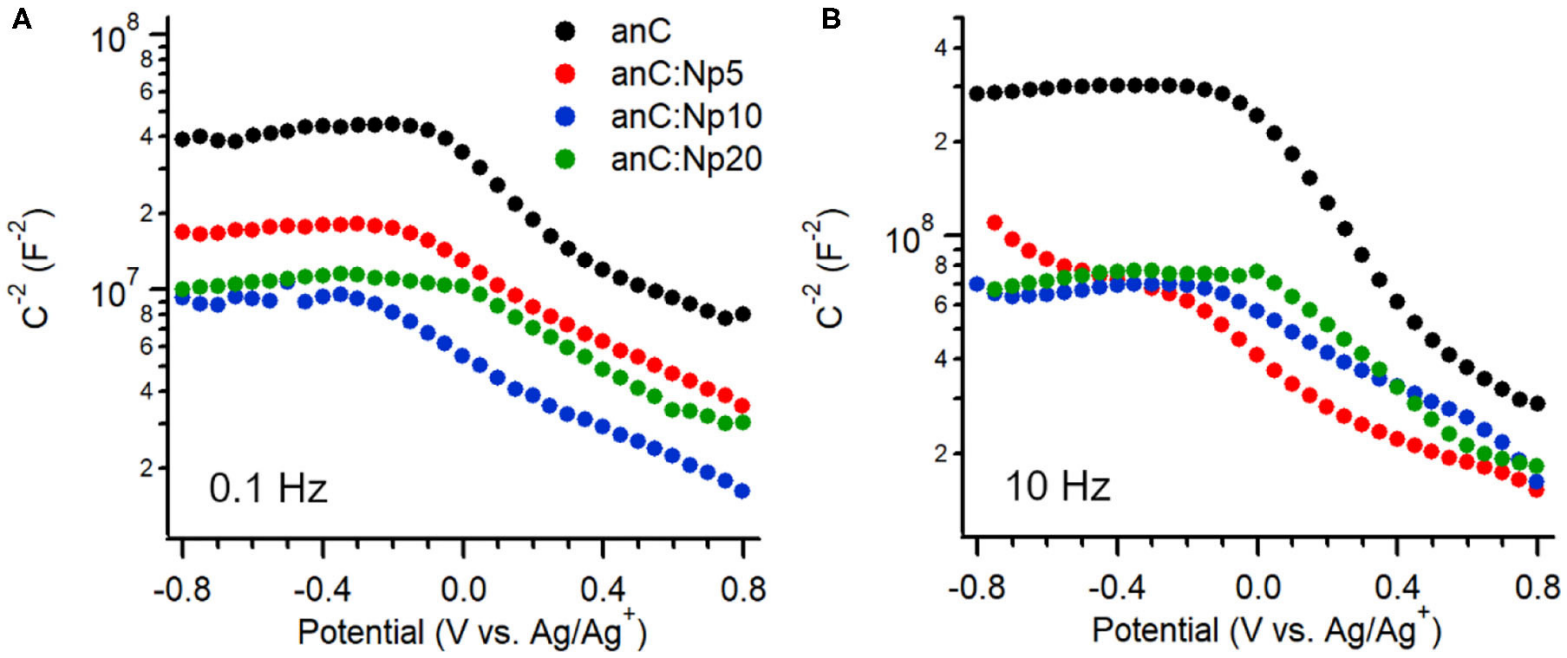
Mott-Schottky plots of anC, anC:Np5, anC:Np10, and anC:Np20 carbon materials obtained in 0.1 M TBAPF6 in acetonitrile at **(A)** 0.1 Hz and **(B)** 10 Hz.

The interfacial capacitance of the electrode materials was investigated as a function of potential in the same electrolyte. Figures 5A,B show Mott-Schottky plots of the equivalent series capacitance obtained at 0.1 Hz and 10 Hz, respectively, at potentials in the range −0.8 to +0.8 V vs. Ag/Ag^+^ (Hoque et al., 2019). The plot of the nitrogen-free anC material shows the characteristic response of a p-type semiconductor under conditions in which the capacitance is dominated by the space charge layer (Memming, 2001). This is in agreement with previous reports of p-type behavior for nitrogen-free amorphous carbon electrodes obtained in either organic or aqueous electrolytes (Colavita et al., 2007; Hoque et al., 2019). Plasma treatment results in a change in capacitance values at 0.1 and 10 Hz, however the p-type character of the material is preserved, indicating that the introduction of N-sites via this surface treatment does not significantly change the semiconducting character. This is in stark contrast to the effect of bulk nitrogen incorporation which instead was shown by our group to result in a capacitive response with greater n-type character (Hoque et al., 2019) in amorphous carbons. The minimum of the interfacial capacitance at 0.1 Hz (see Supporting Information) was found to increase by *ca*. 60% after 5 min exposure, while leveling off at approximately twice the value of the nitrogen-free material after 10 min. This is consistent with the introduction of both N-sites and defects; these are known to result in (i) an increased density of mid-gap localized/surface states (Van Tuan et al., 2012; Zhong et al., 2014; Velický et al., 2019; Toh et al., 2020) which lead to increased interfacial capacitance (Wiggins-Camacho and Stevenson, 2009; Tian et al., 2015; Velický et al., 2019), albeit at the expense of delocalization; and (ii) an increase in electrode roughness and consequently its electrochemical specific surface area (ECSA). A similar increase of the capacitance by a factor of 2 is observed at 10 Hz; notably, a comparison of values obtained at 0.1 vs. 10 Hz indicates dispersion in the capacitive response. This is generally observed in disordered/heterogeneous electrodes (Pajkossy, 1994; Kerner and Pajkossy, 2000), and had been previously reported also in the case of bulk-doped nitrogenated amorphous carbons (Hoque et al., 2019). The absence of a further increase in capacitance when increasing the exposure time from 10 to 20 min, suggests that additional amorphization negatively affects long-range properties and metallic character of the carbon material, in agreement with observed trends following ion bombardment of graphene (Van Tuan et al., 2012; Zhong et al., 2014) and after progressive bulk nitrogenation in amorphous carbons (Behan et al., 2017; Hoque et al., 2019).

### ORR Activity of Plasma-Treated N-Doped Carbons

**Figure 6:**
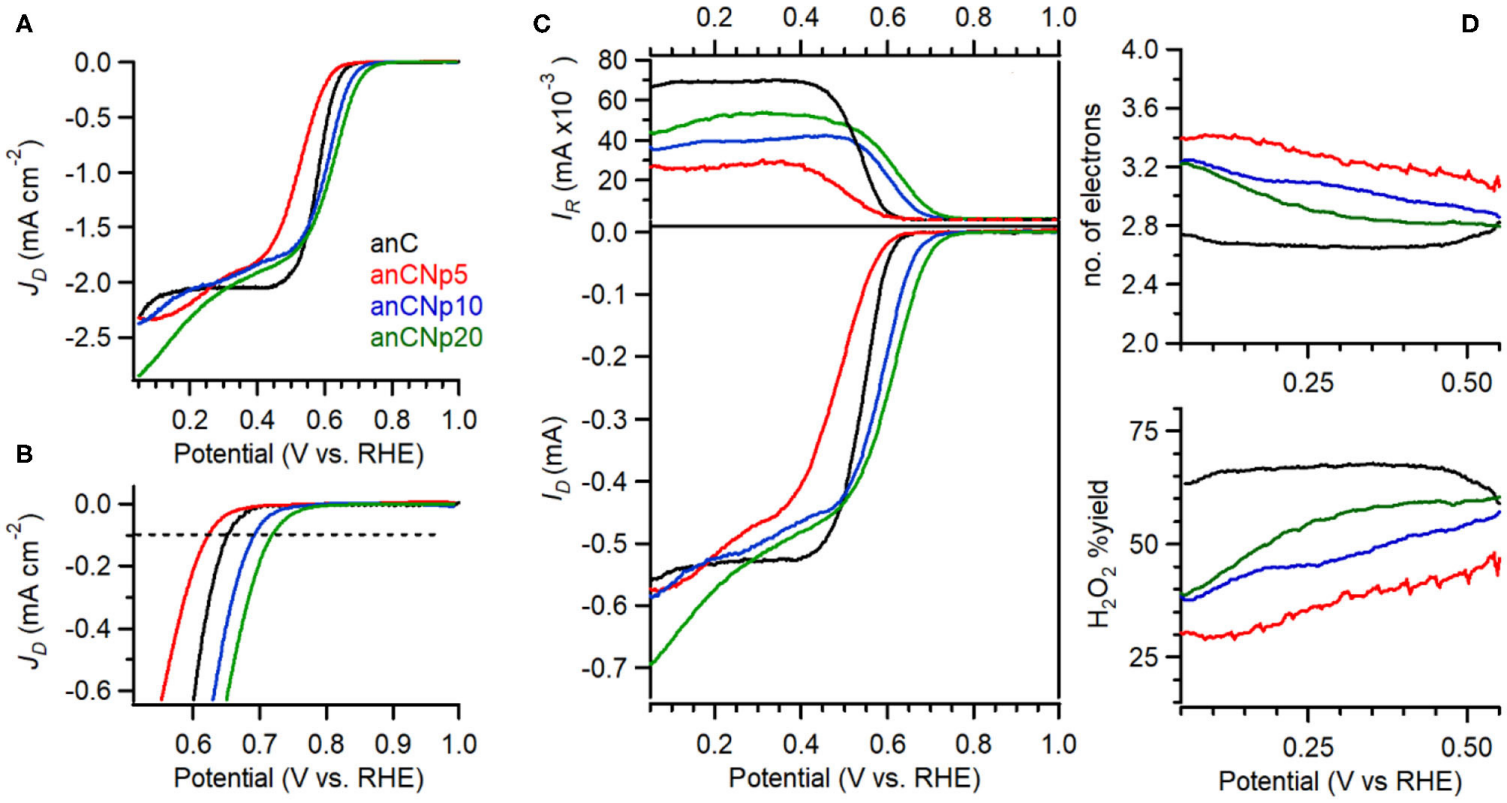
**(A)** RDE voltammetry obtained in O_2_-saturated 0.1 M KOH solutions at 50 mV s^−1^ and 900 rpm for anC, anC:Np5, anC:Np10, and anC:Np20; **(B)** shows details of the onset region including the reference 0.1 mA cm^−2^ current density used to estimate E_on_ values. **(C)** RRDE data showing disk (bottom) and ring (top) currents at 1,600 rpm. **(D)** Calculated H_2_O_2_% yield (bottom) and average number of electrons (top) from data in **(C)**.

The electrocatalytic activity of plasma-treated carbon materials was investigated in O_2_-saturated 0.1 M KOH via RDE methods. Figure 6A shows linear sweep voltammograms (LSV) at 50 mV s^−1^ of anC, anC:Np5, anC:Np10 and anC:Np20; all curves are shown after subtraction of the capacitive current background. The nitrogen-free material shows a clear onset in the cathodic current, associated with the reduction of O_2_ and a well-defined plateau indicating a mass-transport limited current (Behan et al., 2019b). The onset potential (E_on_), defined as the potential at which the current density reaches a value of 0.1 mA cm^−2^, is a useful descriptor to compare electrocatalyst performance (Behan et al., 2019a); values observed for all electrode materials are summarized in Table 3. The nitrogen-free anC electrode yields an E_on_ comparable to that of glassy carbon or undoped carbon nanofiber electrodes (Stamatin et al., 2016). Exposure to nitrogen plasma results, first, in a slight negative shift of E_on_, as shown in greater detail in Figure 6B; however, longer exposures result in a progressive improvement reaching 0.72 V_RHE_ after 20 min of exposure. This indicates that, despite anC:Np5 and anC:Np20 having similar surface N/C concentrations, the greater structural disorder in the carbon matrix observed in anC:Np20 leads to significant improvements in E_on_. Plasma exposure also yields a general increase in the limiting current density, although the mass-transport limited regions of anC:N electrodes show poorly defined plateaus. A non-zero slope in this region is typically observed for electrocatalysts with a distribution of different active sites; its presence in Figure 6A is therefore consistent with an increase in structural disorder upon plasma exposure, also evidenced by Raman and EIS results.

**Table 3:** Summary of key indicators or ORR activity and selectivity obtained for nitrogen-free and plasma-treated carbon electrode materials.

**Sample**	**E_**on**_ (V_**RHE**_) @0.1 mA cm^**−2**^**	**H_**2**_O_**2**_% @0.3 V_**RHE**_**	***n* @0.3 V_**RHE**_**
anC	0.65	67	2.6
anC:Np5	0.62	38	3.2
anC:Np10	0.69	47	3.0
anC:Np20	0.72	57	2.9

The selectivity of plasma-treated anC:N electrodes toward the 4e reduction pathway was investigated using rotating ring disk electrode (RRDE) experiments. The most desirable product of the ORR for energy applications is the one resulting from a 4e transfer, i.e., hydroxide/water; however, a partial 2e reduction to hydroperoxide is also possible, as below:

(1) $$\begin{array}{r} {\text{O}}_{2}+2{\text{H}}_{2}\text{O}+{4\text{e}}^{-}\to 4\text{O}{\text{H}}^{-}{\text{E}}^{{}^{\circ}}=1.229\text{ V vs}.\text{ RHE} \end{array}$$

(Hoare, 1985)

(2) $$\begin{array}{r} {\text{O}}_{2}+{\text{H}}_{2}\text{O}+{2\text{e}}^{-}\to \text{H}{\text{O}}_{2}^{-}+\text{O}{\text{H}}^{-}{\text{E}}^{{}^{\circ}}=0.695\text{ V vs}.\text{ RHE} \end{array}$$

(Hoare, 1985)

In RRDE experiments the Pt ring electrode is used to quantify the amount of hydroperoxide produced at the disk electrode via the anodic reaction corresponding to the reverse of (2). Figure 6C shows plots of ring (top) and disk (bottom) current obtained at 1,600 rpm and 50 mV s^−1^ in 0.1 M KOH. The onset of the ring current mirrors the onset of the disk current in all cases, thus confirming that hydroperoxide detected at the ring arises from the cathodic process taking place at the disk. The differences in ring current reveal significant differences in ORR selectivity at anC:N electrodes. Figure 6D show the calculated yield of hydroperoxide (bottom) and average number of electrons transferred (top) as a function of potential. It is evident from these two figures that for the nitrogen-free anC electrodes the preferred product is hydroperoxide. All three plasma-treated materials show much lower yields of hydroperoxide and higher average number of electrons as reported in Table 3. The differences in selectivity among plasma-treated materials suggest that despite possessing the worst onset, anC:Np5 is the most selective toward the 4e reduction pathway.

It is interesting to compare ORR activity indicators obtained for plasma treated thin film electrodes, with those obtained for bulk N-doped and ammonia-modified thin film electrodes, also synthesized from sputtered precursors and reported in our previous work (Behan et al., 2019a,b). In the case of plasma-treated anC:N, all electrodes were based on a highly graphitized nitrogen-free precursor film that was then modified via low energy plasma, yielding predominantly pyrrolic-N and a progressively greater density of defects. The introduction of N-sites imparts ORR activity, as evidenced by improvements in E_on_, limiting current densities and selectivity, while contributions to the activity by O-sites resulting from plasma treatment might also play a role (Wu et al., 2017) in the observed general improvement. However, N/C and O/C content, as well as functionality composition remain similar across anC:N materials and therefore do not fully explain the observed activity trends. On the other hand, the disorder/amorphization that accompanies plasma modifications appears essential for modulating the activity descriptors. A comparison of onset potentials shows that they follow the order anC:Np5 < anC < anC:Np10 < anC:Np20. It cannot be ruled out that changes in the onset potentials may arise due to microscopic roughness. However, this appears unlikely, as the changes in interfacial capacitance cannot justify the observed E_on_ trend: the minimum capacitance of anC is lower than that of anC:Np5, while that of anC:Np20 samples is smaller than that of anC:Np10. Therefore, we propose that the most probable origin of improved onsets is an increase in the concentration of structural defects, such as edges and bond distortions, which have been proposed to be active sites in the electrocatalysis of the ORR (Favaro et al., 2013; Byers et al., 2014; Jia et al., 2016, 2019) and other reactions of importance for sustainable energy technologies (Dabo et al., 1998; Siahrostami et al., 2017; Kumatani et al., 2019).

Finally, it is interesting to note that despite improvements in E_on_ and values of *n* = 3 resulting from nitrogen plasma treatments, none of the anC:N carbon film electrodes displayed the high E_on_ values (0.83 V_RHE_) and remarkable selectivity (*n* = 4) observed for highly graphitized carbon thin films electrodes with low N/C content but ca. 50:50 ratio of pyridinic-N and graphitic-N (Behan et al., 2019b). This suggests that the N-site composition remains critical for achieving the best performances. Further studies of model electrodes containing exclusively pyrrolic-N and controlled combinations of the same with pyridinic-N and graphitic-N might improve our understanding of synergies between these sites, as previously demonstrated by our group for other N-functionalities (Behan et al., 2019b).

## Conclusion

In this work we used model thin film electrodes based on metal-free sputtered deposited amorphous carbon to investigate the effects of nitrogenation via plasma treatment on the electrochemical and electrocatalytic response of carbon materials. Sputtered and graphitized nitrogen- and metal-free carbon films were used as precursors to prepare model electrodes with different degrees of surface modifications. Plasma treatments result in both chemical and structural changes, as N-sites and C-defect sites were introduced following ion bombardment. Interestingly, chemical modifications occur rapidly and N/C concentration and N-site composition stabilize after relatively short exposure times; however, structural changes progress at slower rate. This provided us an opportunity to understand the individual contributions of chemical and structural changes to the overall electrocatalytic response of the materials in the ORR, an important reaction for sustainable energy technologies.

A combination of spectroscopic and electrochemical methods was used to study the composition and the electrochemical response of the materials in supporting electrolyte and in the presence of O_2_ in alkaline solutions. The introduction of predominantly pyrrolic-N at the carbon surface was found to only slightly affect the electrochemical performance of the materials in the ORR, in the absence of enhancement in structural disorder. It is the combination of N-site and C-defects that appears to have the greatest effects on ORR activity affecting both onsets and selectivity. We attribute this to the availability of a higher density and a wider distribution of active surface sites available to the reaction which is evidenced also in changes in the interfacial properties observed via optical and impedance spectroscopy.

Our results suggest that structural defects in the carbon matrix play an essential role in imparting and modulating electrocatalytic activity at metal-free carbons. The nanostructuring that typically accompanies surface modification reactions is likely as important as the introduction of the localized heteroatom functionality and must be taken into account for the design of carbon electrodes with tailored reactivity.

## Data Availability Statement

The raw data supporting the conclusions of this article will be made available by the authors, without undue reservation.

## Author Contributions

MH prepared the first draft of the manuscript and carried out the majority of experimental work. JC and JL designed and optimized the plasma system used for surface modifications. TP and JB contributed with interpretation of Raman and electrochemical results, respectively. PC contributed the final version of the manuscript, oversight, experimental design, and interpretation. All authors have given their approval to this manuscript.

## Conflict of Interest

The authors declare that the research was conducted in the absence of any commercial or financial relationships that could be construed as a potential conflict of interest.
