# Supplementary material for: Reactive Plasma N-Doping of Amorphous Carbon Electrodes: Decoupling Disorder and Chemical Effects on Capacitive and Electrocatalytic Performance
Source: Front Chem. 2020 Nov 3;8:593932. doi: 10.3389/fchem.2020.593932 (PMC7670066; doi:10.3389/fchem.2020.593932)
Supplement: Supplementary file 1 [file Data_Sheet_1.PDF]

Supporting Information

Reactive plasma N-doping of amorphous carbon electrodes: decoupling disorder and chemical effects on capacitive and electrocatalytic performance

Md. Khairul Hoque,[a] *James A. Behan,*[a] *James Creel,*[a] *James Lunney,*[b] *Tatiana S. Perova*[c] *and Paula E. Colavita*[a1]

a – School of Chemistry, CRANN and AMBER Research Centres, Trinity College Dublin, Dublin 2, Ireland

b – School of Physics, Trinity College Dublin, Dublin 2, Ireland

c - School of Engineering, Trinity College Dublin, College Green, Dublin 2, Ireland

[1] Corresponding author: colavitp@tcd.ie

Text S1. Additional XPS results

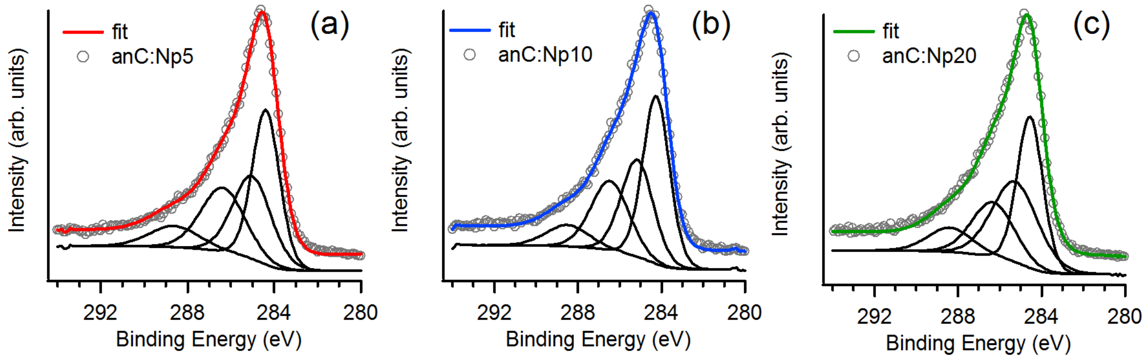

Figure S1. High resolution C 1s spectra of (a) anC:Np5, (b) anC:Np10 and (c) anC:Np20 together with their best-fit deconvolution. Individual peak contributions are offset from the spectral envelope for the sake of clarity.

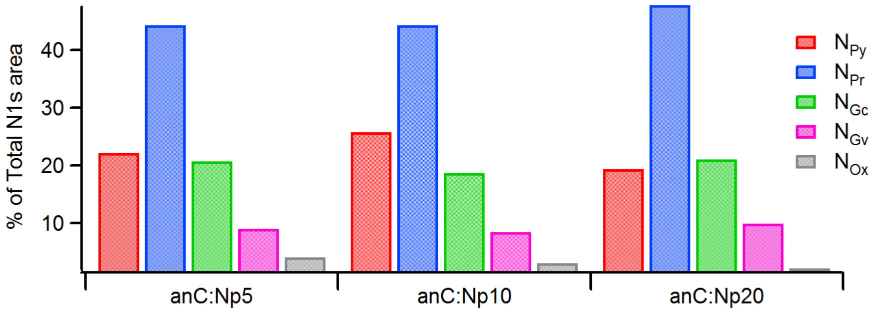

Figure S2. Distribution of N 1s components obtained from best-fits of the high resolution N 1s spectra of anC:Np5, anC:Np10 and anC:Np20 electrodes using five contributions corresponding to pyridinic-N (N_{Py}), pyrrolid-N (N_{Pr}), graphitic-center (N_{Gc}), graphitic-valley (N_{Gv}) and oxides/satellites (N_{Ox}).

Text S2. Additional Raman spectroscopy results

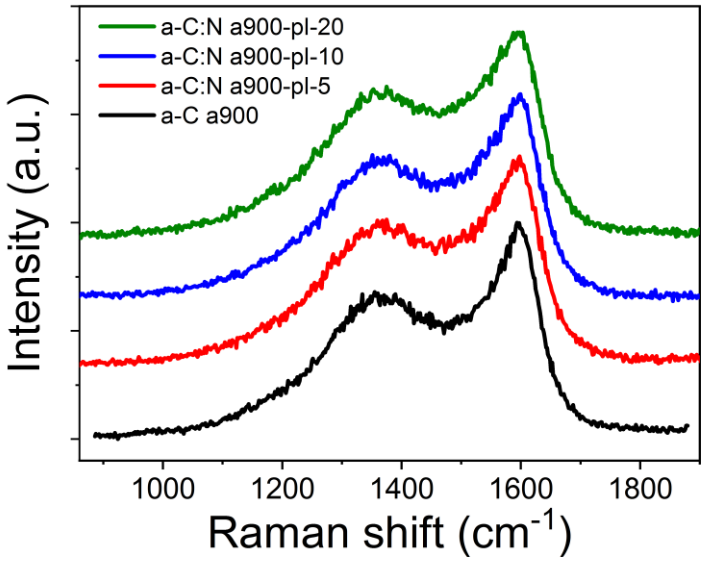

Figure S3. Raman spectra of amorphous carbon electrodes prepared with varying N2% content in the deposition gas mixture; excitation 488 nm. Spectra are normalised relatively to the G band intensity.

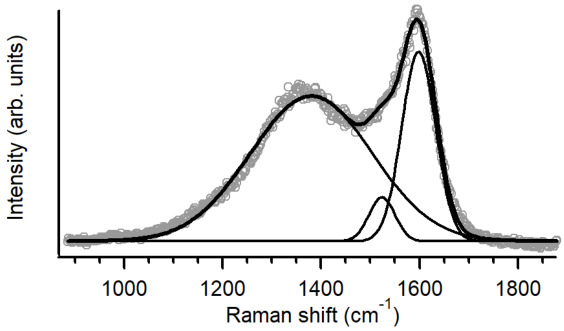

Figure S4. Raman spectrum of N-free anC materials and its fitting using 3 contributions; excitation 488 nm. Reproduced from data in (Behan et al. *J. Phys. Chem. C* **2018,** *122*, 20763).

Text S3. Additional capacitance results

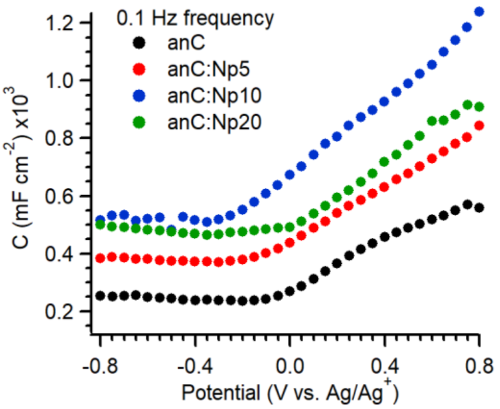

Figure S5. Interfacial capacitance obtained from EIS experiments in 0.1 M TBAPF6 in acetonitrile at 0.1 Hz and normalization by geometric area.

Table S1. Geometrical roughness factor *R* calculated from a ratio of the minimum capacitance at 0.1 Hz, normalized by the capacitance of a polished GC electrode measured under the same conditions (C_{dl}/C_{GC}), under the assumption that roughness changes alone are responsible for changes in C_{dl} values.

	R
**anC**	7.9
**anC:Np5**	12
**anC:Np10**	17
**anC:Np20**	15
